# Clinical Outcomes Comparing Two Prosthetic Knee Designs in Individuals with Unilateral Transfemoral Amputation in Turkey

**DOI:** 10.33137/cpoj.v4i1.35297

**Published:** 2021-06-29

**Authors:** A Yazgan, S Kutlutürk, K Lechler

**Affiliations:** 1 Orthotics - Prosthetics Master of Science Program, Graduate School of Healthy Sciences, Istanbul Medipol University, Istanbul, Turkey.; 2 Össur Turkey Academy, Istanbul, Turkey.; 3 Department of Physical Therapy and Rehabilitation, School of Healthy Sciences, Istanbul Medipol University, Istanbul, Turkey.; 4 Össur Medical Office, R&D Össur ehf, Reykjavik, Iceland.

**Keywords:** Balance, Functional Mobility, Prosthetic Satisfaction, Transfemoral Amputation, Quality of Life, Prosthesis

## Abstract

**BACKGROUND:**

Clinical outcome assessments provide important input for the rehabilitation of individuals with transfemoral amputation. Differences in prosthetic knee designs may influence clinical outcomes.

**OBJECTIVE(S):**

The aim of this study was to compare functional mobility, balance, prosthetic satisfaction and quality of life in individuals with unilateral transfemoral amputation with microprocessor-controlled (MPK) and non-microprocessor knee designs (Non-MPK).

**METHODOLOGY:**

The study included ten experienced MPK (Rheo Knee) users (Group 1) and ten experienced Non-MPK (Total Knee^®^ 2000) users (Group 2). For mobility; the 6 Minute Walk Test (6MWT), for balance; the Berg Balance Scale (BBS), Single Leg Stand Test (SLST) and Four Square Step Test (FSST), for quality of life; the Nottingham Health Profile (NHP) and for prosthetic satisfaction; the Satisfaction with Prosthesis Questionnaire (SATPRO) were administered.

**FINDINGS:**

6MWT results of the MPK group were significantly higher than Non-MPK group (p<0.05). In the MPK group a strong negative correlation was found between the FSST and the 6MWT (r=-0.661, p=0.038). No statistically significant differences were found between the groups (p>0.05) comparing balance, prosthesis satisfaction and quality of life values.

**CONCLUSION:**

The findings will inform about the patient’s prognosis and the expected clinical outcomes when prescribing an MPK or an Non-MPK. Individuals with unilateral transfemoral amputation covered longer distances using an MPK compared to Non-MPK.

## INTRODUCTION

Lower extremity amputation causes impairment in functional mobility, sensation, body image and quality of life. As the functional mobility decreases, quality of life (QoL) decreases and the risk for joint degradation increases.^1,2^ Typical challenges encountered in rehabilitation of individuals with transfemoral amputation (ITF) are reduced walking distances,^3^ balance impairment^4^ and increased metabolic cost ^5^ Adequate prosthetic component selection for ITF is a critical factor to assist in prevention of additional burden and the restoration of functional mobility. ^6^ A large variety of prosthetic knee designs are available for individuals with transfemoral amputation. ^7^

The appropriate prescription requires comprehensive consideration to provide safety, satisfaction and function, ^8^ and therefore relies on qualified professionals to select the adequate solution. In line with theses objectives, the development of prosthetic knees aims to create a device that provides balance confidence, balance ability and to support walking performance in ITF. ^9^ Historically, transfemoral prostheses use a passive, mechanical mechanism to control the swing and stance phases of the prosthetic gait. Today, microprocessor controlled knees (MPK) are becoming more common. ^10^ Even though Non-MPK and MPK are functionally similar in range of motion acting, within one degree of freedom, MPK designs allow dynamic management of the motion behavior throughout the gait cycle. ^8^ The sensor technology allows for quicker adaptation to varying walking speeds by making decisions on the application of resistance and appropriate transitions. ^11^

Walking with an MPK has been reported to improve gait symmetry, ^12^ assist in more physiological mobility and loading pattern on the prosthetic side, ^13^ and reduce loading on the contralateral side.^14^ Some clinical studies are inconclusive,^14,15^ others report clear differences between the MPK and Non-MPK in functional performance, balance and psychology parameters.^16^

The majority of literature comparing functional outcomes have been conducted in Western societies and rarely among Turkish ITF. Differences in culture or rehabilitation practices may affect these outcomes.

The aim of the study was to compare balance, functional mobility, prosthetic satisfaction and QoL in individuals with unilateral transfemoral amputation using Non-MPK versus MPK after long term adaptation in Turkey. We hypothesized that there is a difference between individuals using MKP and Non-MPK in balance, functional mobility, prosthetic satisfaction and quality of life.

## METHODOLOGY

A two group design was chosen to compare objective performances, observer-reported and patient-reported outcomes after longterm adaptation on the prescribed MPK; (Group 1) Rheo Knee (Össur, Reykjavik, Iceland) and (Group 2) Non-MPK; Total Knee^®^ 2000 (Össur, Reykjavik, Iceland). For consistency and to reduce confounding factors all participants were recruited from one clinic. All twenty subjects used the energy storing and-return (ESAR) Vari-flex^®^ 2000 (Össur, Reykjavik, Iceland) foot design.

The study was approved by Istanbul Medipol University ethics institutional review board, and informed written consent was obtained. The criteria for enrollment included unilateral transfemoral amputation, age between 18-59 years and a minimum of one year experience with the same prosthesis, Medicare functional classification level K3 or K4, ^17^ and the ability to walk at least 10 m without using any assistive device. Criteria for exclusion were chronic residual limb skin breakdown. Subjects were also excluded if they had an acute illness, chronic illness, dependency on walking aids, neuromuscular problems or any secondary medical condition that preclude performance of the test protocol. The subject profile data is presented in **Table 1**.

**Table 1:** Participant details

	Group 1 (MPK) (n=10)		Group 2 (Non-MPK) (n=10)		Mann Whitney U Test		
	Min-Max	Mean±SD	Min-Max	Mean±SD	u	z	p
**Age**	23.0-50.0	38.0±8.4	18.0-59.0	39.3±13.0	49.00	-0.07	0.97
**BMI (kg/m^2^)**	19.0-26.9	23.7±2.6	16.9-29.7	24.3±4.1	46.00	-0.30	0.79
**Height (cm)**	170.0-183.0	177.6±4.4	150.0-191.0	170.1±13.2	32.00	-1.36	0.19
**Weight (kg)**	55.0-90.0	75.0±10.6	46.0-88.0	70.1±13.7	39.50	-0.79	0.43
**Time of amputation (years)**	3.0-37.0	22.5±11.1	1.5-31.0	14.1±12.7	31.00	-1.43	0.16
**Prosthetics use experience (years)**	2.0-35.0	18.9±9.7	1.0-31.0	13.1±12.3	34.50	-1.17	0.24
**Experience with Non-MPK/MPK (years)**	1.0-8.0	5.7±2.9	1.0-10.0	3.7±3.2	31.00	-1.46	0.16
**Daily standing time (hour)**	1.0-16.0	8.8±4.7	2.0-15.0	7.4±5.0	40.50	-0.72	0.48
**Daily exercise time (hour)**	1.0-4.0	2.1±1.4	1.0-4.0	2.1±1.2	43.00	-0.55	0.63

Min: Minimum, Max: Maximum, SD: Standard Deviation, p value is significant when p< 0.05.

Balance was evaluated using the BBS,^18^ FSST^19^ and SLST.^20^ The measures were administered in the same order for all the subjects starting with the BBS. The 14 items of the BBS were answered by the participants with the supervision of researchers only assisting when the participant asked for clarification. Following this the observational section of the BBS test with its 5 point ordinal scale (0-4) was administered. The maximum score that can be achieved is 56, with higher scores reflecting better balance. A score of 45 is required for independent safe ambulation.^18^ Following the BBS, static balance was assessed by the SLST. With a 5 minute break between each task single leg standing was timed on the amputated and non-amputated side. Participants started on the non-amputated side inside a parallel bar. The time the participants stood on one leg was measured with a stopwatch and recorded in "seconds". The stopwatch was started as the participants raised the contralateral leg and removed the hands or any support from the side bars. Time was stopped when the participants reached out for support or contacted the ground with the contralateral side or by reaching the maximum standing time of 30 seconds.^20^

For the FSST individuals were asked to step across four squares, as quickly as possible following the instructions provided by Gouelle et. al. 2020.^19^ Participants had to step forward, backward, right, and left and the time of completion was noted. The FSST was repeated two times and the second result was reported.

The 6MWT was administered according to the instructions of the American Thoracic Society^21^ in order to capture the walking distance covered by the participants within the 6 minutes given. The 30 meters walkway was solid and leveled and marked every 3 meters. Pylons marked the end points. Participants were instructed to start walking when ready and follow the researcher's instructions and reminded not to run or jog. As soon as the participants started to walk the timer was started. With the sound of the timer the subject stopped walking and sat down on a chair. The distance covered within 6 minutes was recorded.

The Nottingham Health Profile (NHP) and the Satisfaction with Prosthesis Questionnaire (SATPRO) were administered in a paper based format in Turkish.^22,23^ All measures were conducted within half a day and paper-based results were scanned and transferred to a digital format.

### Statistical analysis

Using an effect size of 1.629 based on previously reported data for BBS, 6MWT, NHP and SATPRO,^23^ a sample size of 18 (9 per group) was deemed necessary to ensure a type-1 error rate of 0.05 and power of 0.95. To account for drop outs, the study was conducted with the participation of 20 transfemoral individuals with amputation. All analyses were performed using SPSS version 21.00 (IBM, USA). The p value was set to 0.05 in all statistics. The Mann Whitney U test was used to detect group differences. The relationship between FSST and 6MWT was evaluated using Pearson Correlation.

## RESULTS

Twenty ITF were recruited at a single study site. No differences in age, height, weight, body mass index, time since amputation, prosthetic experience, experience with MPK/Non-MPK, patient-reported daily standing and daily exercise time between groups (p> 0.05) could be identified (**Table 1**, **Table 2**).

**Table 2:** Participant gender, side of amputation, cause of amputation and residual limb length.

		Group 1 (MPK)	Group 2 (Non-MPK)
**Gender**	**Female**	1(10%)	3(30%)
	**Male**	9(90%)	7(70%)
**Amputation Side**	**Right**	5(50%)	7(70%)
	**Left**	5(50%)	3(30%)
**Cause of amputation**	**Trauma**	8(80%)	4(40%)
	**Congenital Abnormalities**	1(10%)	3(30%)
	**Peripheral Vascular Disorder**	1(10%)	1(10%)
	**Cancer**	0(0%)	1(10%)
	**Infections**	0(0%)	1(10%)
Residual Limb Length^*^	**Short**	1(10%)	2(20%)
	**Medium**	7(70%)	4(40%)
	**Long**	2(20%)	4(40%)

^*^ as defined in the standard ISO/WD 8548-2:2018(E): amputation level= short (proximal third)/medium (middle third)/long (distal third)

There were no differences between two groups for the BBS, SLST and FSST (p> 0.05) (**Table 3**). All participants achieved the maximum of 30 seconds for the SLST on the sound side.

**Table 3:** Comparison of the balance parameters of two groups.

	Group 1 (MPK)		Group 2 (Non-MPK)		Mann Whitney U Test		
	Min-Max	Mean±SD	Min-Max	Mean±SD	*u*	p	z
**SLST(sec)**	0.0-5.0	1.7±2.0	0.0-7.0	0.8±2.2	35.00	0.28	-1.33
**FSST(sec)**	2.6-6.3	4.9±1.2	4.0-7.8	5.4±1.3	44.00	0.68	-0.45
**BBS**	50.0-54.0	52.5±1.7	46.0-55.0	51.4±2.8	-1.01	0.35	37.00

SLST: Single Leg Stand Test prosthetic side, FSST: Four Step Square Test, BBS: Berg Balance Scale, Min: Minimum, Max: Maximum, sec.: second, SD: Standard Deviation, p value is significant when p<0.05.

There were no differences between the two groups for the SATPRO and NHP scores (p> 0.05) (**Table 4**). NHP sub-parameters range from 0 to 100. Higher NHP scores relate to more pain, more social isolation, more emotional reaction but less physical abilities, energy level and sleep.

**Table 4:** Comparison of quality of life (NHP) and patient satisfaction (SATPRO).

		Group 1 (MKP)	Group 2 (Non-MPK)	Mann Whitney U Test		
		Mean±SD	Mean ±SD	*u*	*z*	p
**Patient satisfaction (SATPRO)**		33.3±5.2	35.7±2.7	39.50	-0.80	0.43
Quality of life (NHP subtests)	**Pain**	6.5±7.8	11.7±15.8	41.50	-0.67	0.52
	**Social Isolation**	0.0±0.0	3.5±11.2	45.00	-1.00	0.73
	**Emotional Reaction**	1.0±3.3	1.8±5.5	49.50	-0.07	0.97
	**Physical Abilities**	4.4±7.7	9.7±11.9	39.00	-0.94	0.43
	**Energy Level**	11.0±17.8	11.0±17.8	50.00	0.00	1.00
	**Sleep**	1.3±4.0	0.0±0.0	45.00	-1.00	0.73
	**Total Score**	24.2±27.8	37.8±36.0	44.50	-0.88	0.39

Min: Minimum, Max: Maximum, SD: Standard Deviation, p value is significant when p< 0.05.

A statistically significant difference was found between two groups for the 6MWT distance (p< 0.05). 6MWT results of Group 1 were statistically significantly higher than Group 2 (p< 0.05) (**Table 5**).

**Table 5:** Comparison of 6MWT distance of two groups.

	Group 1 MKP		Group 1 Non-MPK		Mann Whitney U Test		
	Mean±SD	Min-Max	Mean±SD	Min-Max	*u*	z	p
**6 MWT (m)**	474.8±56.1	386.0-552.0	346.6±60.5	276.0-445.0	7.00	-3.25	0.00*

6MWT: 6 Minute Walk Test, SD: Standard Deviation m: meter, p value is significant when p< 0.05.

For Group 1, a high negative correlation was found between the FSST and 6MWT (p< 0.05) (**Table 6**).

**Table 6:** Correlation between 6MWT, FSST, BBS, SLST and residual limb length within the two groups.

	Group 1 (MKP)		Group 2 (Non-MPK)	
	6MWT (m)		6MWT (m)	
	r^*^	p^*^	r^*^	p^*^
**BBS**	0.20	0.66	0.39	0.71
**SLST (sec)**	0.37	0.37	0.54	0.06
**FSST (sec)**	-0.66*	0.02*	-0.40	0.22
Residual Limb Length (short/medium/long)^**^	0.17	0.62	0.42	0.22

6MWT: 6 Minute Walk Test, BBS: Berg Balance Scale, FSST: Four Square Step Test, SLST: Single Leg Stand Test, m: meter, sec: second,

^*^ Spearman Correlation Test, Statistical significance limit is 0.05, r: Spearman’s rank correlation coefficient.

^**^ according to ISO/WD 8548-2:2018(E): amputation level= short (proximal third)/medium (middle third)/long (distal third).

## DISCUSSION

Our study compared two groups of ITF (MPK and non-MPK) who had been using their prosthesis between 1 and 10 years. The results indicate that subjects in the MPK group walked further compared to subjects using a Non-MPK. No difference between the groups was found for patient reported balance, prosthesis satisfaction and QoL. In addition, a high correlation was found between the dynamic balance and functional activity of ITF using MPK.

The parallel group design allowed us to avoid order affects that have been reported in previous studies.^24,25^ In addition, previous studies using a randomized cross over design have had shorter adaptation and as a result failed to demonstrate that subjects reached a plateau in their performance measures.^17^ By contrast a minimum usage time of one year makes it highly likely that performance plateau was reached for the subjects in this study.

Balance is beneficial for daily functioning of patients with a lower limb amputation and often assessed by the SLST ^20^ to reliably test the balance performance. It is known that SLST is less on the amputated side than on the unaffected side. Comparing the two groups by SLST, no statistically significant difference between the Non-MPK and the MPK could be detected on the prosthetic side.

Major et. al.^18^ reported a BBS median score of 52 (49-54) for ITF and showed high reliability and validity of BBS in community-dwelling persons with lower extremity amputation. Despite not being specific to prosthetic knees, differences in BBS score of 49.0± 9.9 were found^26^ between mechanical and hydraulic (non-MPK) stance controlled knees. Also, BBS showed sensitivity when transitioning from the Non-MPK to MPK (Rheo Knee) with an increase for MPK users scoring between 54 and 56. ^15^ Both studies reported on BBS shortly after transitioning. In our study, no statistically significant difference in BBS scores was found after a long adaptation period. The BBS scores (52.50 ± 1.26) being generally high may indicate that users have adapted well and the BBS scores were not sensitive to differences in knee component designs for individuals having used them for a long time.

Lythgo et. al.,^27^ examined the function, gait and dynamic balance of ITF using two different Non-MPK designs. There was no difference in FSST (13.6±3.0 / 13.2±2.2 seconds) for the two Non-MPK. Our findings showed 4.85 seconds in FSST for MKP and 5.44 seconds for Non-MPK. Unlike the literature^19, 27, 28^ both groups in our study performed almost 1sec faster than Kahle et. al. 2016^29^ indicating a generally higher dynamic balance level. Kahle et. al. compared two socket technologies and their volume adaptation on one single user. Our cohort included high active users, e.g. playing soccer, dancing, etc. which likely explains their improved performances. The small difference in favor of the Non-MPK may be explained by the higher ratio of congenital amputation cause and longer residual limb length in the Non-MPK group. Kamali et. al.^30^ indicated that a longer residual limb may improve standing stability and dynamic balance.

Hafner et. al.^8^ showed that the transition from Non-MPK to MPK significantly increases function and performance. In our study there was a significant difference between the two groups in walking distance covered during the 6MWT. In contrast to the aforementioned studies that showed performance difference after only a couple of weeks adaptation time, our study included two different groups and showed these effects after a long adaptation period of several years.

Using the NHP, Demet et. al. assessed the importance of different factors associated with health related QoL in LLAs.^31^ They found physical disability, pain and energy level^32^ to be the most important factors and mobility to be the only independent factor for health related QoL measured by NHP. Ülger et. al.^26^ found a total NHP score of 91.1±28.6 and 51.9±12.5 for two different Non-MPK in contrast to higher scores in our study for both Non-MPK and MPK of 37.77±35.97 and 24.21±27.82, respectively. Burçak et. al.^33^ reported on increased quality of life, improved functional performance, increased prothesis satisfaction and decreased perception of body image disturbance when using an MPK prosthesis versus a Non-MPK. They used the SATPRO questionnaire for prosthetic satisfaction and observed an increase in the use of MKP. We found no difference in Qol and prosthesis satisfaction between the two groups.

Azuma et. al.^34^ found a correlation between dynamic balance and walking ability in ITF using the BBS and 6MWT. Similarly, our study supports the relationship between balance parameters and functional activity. Comparing both groups the MPK showed a high negative correlation between 6MWT and FSST. ITF using an MPK demonstrated good dynamic balance and increased functional activity.

### Limitations

The limitations in this study include the recruitment constraints, resulting in a small sample size and therefore the results cannot be generalized. Although small samples limit generalizability of the results, small samples are common in rehabilitation research^35^ and they relate to identification, recruitment, and enrollment of subjects. The two group design is lacking the statistical strength of a paired design. In our study, balance, functional mobility, prosthesis satisfaction and QoL of ITF using MPK and Non-MPK were compared in two similarly profiled groups with long-term accommodation to their prescribed prosthesis. A cross over design with such a long adaptation time would be unfeasible. In future studies we would like to include more subjects to show differences in balance and mobility with MKP and Non-MPK after long-term adaptation. We propose that in future studies, more ITF using different types of prosthetic knee design should be included to provide more information for practitioners.

Outcomes were only measured at one time point, and thus might only reflect the specific situation at the time of measurement. Longitudinal designs allow statement on reliability not possible herein. No objective balance measures were used to add strength and sensitivity to the findings for the patient and observer-reported measures. The inclusion of only one type of MPK (Rheo Knee) and Non-MPK (Total Knee^®^ 2000) does not allow to generalize these results to other prosthetic knee designs. However limiting the number of knee designs allowed for the identification of the specific outcomes related to the designs.

## CONCLUSION

While this study did not highlight differences in most of the clinical outcomes administered, subjects using an MPK were able to walk longer distances in comparison to those using a Non-MPK. These findings will provide decision makers with a more accurate prognosis when selecting between the two knee designs.

## DECLARATION OF CONFLICTING INTERESTS

The authors had no interests which might be perceived as posing a conflict or bias.

## AUTHOR CONTRIBUTION

- **Ayse Yazgan:** Contributed to the study concept and design, participated in data gathering, analyzed and interpreted data, contributed to the drafting of the manuscript, read and approved the final manuscript.
- **Seval Kutlutürk:** Contributed to the study concept and design, analyzed and interpreted data, contributed to the drafting of the manuscript, read and approved the final manuscript.
- **Knut Lechler:** Analyzed and interpreted data, contributed to the drafting of the manuscript, read and approved the final manuscript.

## SOURCES OF SUPPORT

Össur Medical Office, R&D Össur ehf, Reykjavik, Iceland.

## ETHICAL APPROVAL

The study was approved by Istanbul Medipol University ethics institutional review board, and informed written consent was obtained.
